# Association between post procedural parenteral anticoagulation therapy and clinical outcomes in ST-elevation myocardial infarction patients with high thrombotic risk

**DOI:** 10.3389/fphar.2026.1747506

**Published:** 2026-09-22

**Authors:** Ping Lai, Yining Dai, Minggang Yin, Yeshen Zhang, Zijing Li, Xiaoqun Ning, Yupeng Yang, Yu He, Litao Wang, Ning Tan, Ling Xue, Chongyang Duan, Pengcheng He, Yuanhui Liu

**Affiliations:** 1 Department of Cardiology, First Affiliated Hospital of Gannan Medical University, Key Laboratory of Prevention and Treatment of Cardiovascular and Cerebrovascular Diseases, Ministry of Education, Gannan Medical University, Ganzhou, China; 2 Department of Cardiology, Guangdong Provincial People’s Hospital (Guangdong Academy of Medical Sciences), Southern Medical University, Guangzhou, China; 3 State Key Laboratory of Multi-Organ Injury Prevention and Treatment, Department of Biostatistics, School of Public Health, Southern Medical University, Guangzhou, China; 4 The Second School of Clinical Medicine, Southern Medical University, Guangzhou, Guangdong, China; 5 Department of Cardiology, The Second Affiliated Hospital, School of Medicine, Zhejiang University, Hangzhou, China; 6 School of Medicine, South China University of Technology, Guangzhou, China

**Keywords:** all-cause death, bleeding, high thrombotic risk, parenteral anticoagulation therapy, percutaneous coronary intervention, ST-elevation myocardial infarction

## Abstract

**Background:**

Current guidelines advise against routine use of parenteral anticoagulation therapy (PACT) for ST-segment elevated myocardial infarction (STEMI) patients after percutaneous coronary intervention (PCI). However, the effectiveness of PACT in such patients with high thrombotic risk remains uncertain. To determine the impact of PACT on STEMI patients with high thrombotic risk.

**Methods:**

We enrolled STEMI patients with high thrombotic risk from four Chinese hospitals between 1 January 2010, and 31 December 2022. STEMI patients with high thrombotic risk were categorized them into PACT and non-PACT groups. We used multivariate, propensity score, and subgroup analyses to examine the relationship between PACT and clinical outcomes. The primary outcomes were in-hospital all-cause death and in-hospital major bleeding.

**Results:**

Out of 2,001 PCI patients, 473 received PACT. PACT was not associated with a lower all-cause death (adjusted OR, 0.70; 95% CI, 0.33–1.48; P = 0.349) and was not associated with an increased risk of major bleeding (adjusted OR, 0.84; 95% CI, 0.32–2.18; P = 0.720). After a median follow-up of 1.72 years, PACT did not significantly affect all-cause death and major adverse clinical events compared to non-PACT, as confirmed by propensity score and subgroup analyses.

**Conclusion:**

PACT was not statistically associated with a reduced risk of in-hospital all-cause death and an increased risk of major bleeding in STEMI patients with high thrombotic risk.

## 1 Introduction

Primary percutaneous coronary intervention (PCI) and antiplatelet therapy are Class I recommended treatments for patients with ST-elevation myocardial infarction (STEMI), significantly reducing the risk of major adverse cardiovascular events (Ibanez et al., 2018). However, adverse thrombotic events, such as stent thrombosis and repeat revascularization, remain risks after PCI, especially in patients with high thrombotic risk (e.g., those with diabetes, multivessel disease, or chronic kidney disease) (Zhao et al., 2021). Although anticoagulant therapy during PCI effectively prevents thrombotic complications, its benefits post-PCI are uncertain.

Previous studies have shown that routine use of parenteral anticoagulation therapy (PACT) after PCI is associated with higher rates of bleeding without any reduction of ischemic events (Ducrocq et al., 2016; Madhavan et al., 2017; Yan et al., 2024). Current guidelines recommend against routine use of PACT after PCI, unless indicated for conditions for anticoagulation such as atrial fibrillation (Ibanez et al., 2018). Contrarily, recent studies suggest PACT may reduce mortality without increasing major bleeding in STEMI patients after PCI (Yan et al., 2022; Chinese expert consensus on anti, 2024).

Given the conflicting evidence, identifying optimal STEMI patients for anticoagulation therapy is crucial for reducing ischemic events (Gragnano and Calabro, 2022; Chiarito et al., 2018). Prior studies have reported that patients with high thrombotic risk face early post-PCI ischemic events, with 6% developing stent thrombosis and 21% requiring repeat revascularization (Guo et al., 2021). Nevertheless, it remains unclear whether an intense antithrombotic therapy with PACT after revascularization provides additional clinical benefits in those patients. Therefore, the present study aims to evaluate the impact of PACT on clinical outcomes in STEMI patients with high thrombotic risk.

## 2 Methods

### 2.1 Design of the study and involved patients

This multicenter observational cohort study involved 5,335 patients from four Chinese hospitals, enrolled between 1 January 2010, and 31 December 2022. STEMI was defined based on the European Society of Cardiology (ESC) guidelines (Ibanez et al., 2018). High thrombotic risk was defined according to the TWILIGHT criteria (Mehran et al., 2019), requiring at least one clinical feature (age ≥65 years, female sex, acute coronary syndrome with positive troponin, treated diabetes mellitus, or chronic kidney disease [eGFR <60 mL/min/1.73 m^2^]) and at least one angiographic feature (multivessel coronary artery disease, total stent length >30 mm, thrombotic target lesion, bifurcation lesion treated with two stents, or obstructive left main or proximal left anterior descending artery). This definition was applied retrospectively for cohort selection. Patients who had received parenteral anticoagulation treatment before PCI, had an intra-aortic balloon pump or hemodialysis, or had other conditions such as new onset atrial fibrillation during the hospitalization or left ventricular thrombus, and lacked data of parenteral anticoagulation or high thrombotic risk criteria were excluded. The study protocol was approved by the central ethics committee of Guangdong Provincial People’s Hospital and the participating hospitals.

### 2.2 Data extraction and processing

Trained study coordinators extracted all relevant data from the medical records. We collected information on clinical characteristics, cardiovascular risk factors and comorbidity, and laboratory examination on admission. Details of PCI procedures and antithrombotic therapy were also collected, including antiplatelet therapy dosages, dates, and durations of therapy. An independent clinical events committee assessed all adverse clinical events with the treatment details blinded. Two researchers documented details on medical treatments and clinical events, with any discrepancies decided and resolved by a third researcher. Additionally, the remaining data was validated through random audits of the medical records.

### 2.3 Treatment and procedure

PACT administration was determined by the clinicians based on patients’ characteristics and their clinical judgment regarding both bleeding and ischemic risks. Clinicians tended to prescribe PACT for patients who have higher procedural complexity or residual thrombotic burden or those with slow-flow or no-reflow phenomena. Patients were categorized into the PACT group and the non-PACT group, based on whether they received PACT after PCI. The clinical team determined the type and duration of PACT, opting for either low-molecular-weight heparin or fondaparinux. The type and duration of PACT was prescribed at the discretion of the clinicians. Patients received either fondaparinux, 2.5 mg, subcutaneously once daily or low-molecular-weight heparin, 1 mg/kg, subcutaneously twice daily (dosage was reduced to 1 mg/kg once daily among patients with creatinine clearance <30 mL/min) (Yusuf et al., 2006; Chen et al., 2019). The PACT was initiated at least 6 h after PCI and continued for at least 1 day or until discharge. During PCI, unfractionated heparin was administered as the standard PACT in accordance with current guidelines (Levine et al., 2013), given as a bolus dose of 70–100 U/kg. Antithrombotic therapy and other medications were prescribed according to the doctors’ discretion, in line with current guidelines.

### 2.4 Outcomes

The primary outcomes were in-hospital all-cause death and in-hospital major bleeding. Secondary outcomes included in-hospital major adverse clinical events (MACEs), which encompassed all-cause death, re-infarction, target vessel revascularization, and stroke; in-hospital net adverse clinical events (NACEs), defined as a composite of in-hospital MACEs and major bleeding; in-hospital any bleeding, and follow-up outcomes were defined as a composite of all-cause death and stroke during the post-discharge follow-up period. Major bleeding was defined according to the Bleeding Academic Research Consortium (BARC) criteria (grades 3–5) (Kikkert et al., 2014; Mehran et al., 2011), which includes intracranial bleeding, retroperitoneal bleeding, a decrease in hemoglobin of ≥4 g/dL, transfusions required for significant bleeding, and instances necessitating surgical intervention. Any bleeding was defined as a combination of intracranial bleeding, retroperitoneal bleeding, bleeding at the access site, gastrointestinal bleeding, skin or mucosal bleeding, bleeding at other locations, and a hemoglobin drop of ≥3 g/dL.

### 2.5 Statistical analyses

Continuous variables with a normal distribution were reported as mean ± standard deviation, whereas non-normally distributed data were described using median and interquartile range. Group differences were analyzed using the t-test or the Kruskal–Wallis test, based on the data distribution. Categorical variables were expressed as counts and percentages, with comparisons between groups conducted using the chi-square test.

To examine the relationship between PACT and clinical outcomes, both univariate and multivariable analyses were performed. Propensity score matching was performed as a sensitivity analysis to confirm the robustness of the primary multivariable analyses conducted in the entire cohort. Variables that were significant in the univariate analysis or considered clinically relevant were included in the multivariable models.

To account for potential immortal time bias, a sensitivity analysis of in-hospital outcomes was performed using Cox models with PACT as a time-varying covariate. Time zero was defined as completion of the index PCI procedure. Using a counting-process structure, person-time before the first PACT dose was classified as unexposed and person-time thereafter as exposed; patients who never received PACT contributed only unexposed person-time. For each outcome, patients were followed from completion of PCI until the occurrence of that outcome or hospital discharge, whichever came first.

For propensity score matching analysis, patients were matched in a 1:1 ratio (PACT group vs. non-PACT group), with a standardized difference of less than 10% indicating negligible differences in covariates between the groups. All factors listed in Table 1, except length of hospital stay, were considered in developing the propensity score model. For evaluating long-term clinical outcomes, univariate analyses were carried out using the log-rank test, and multivariable analyses were executed with the Cox proportional hazards regression model.

**TABLE 1:** Baseline characteristics of different groups.

Variables	All patients (N = 2001)		*P* value	Propensity-matched patients		*P* value	Standard difference (%)
	Non-PACT (N = 1,528)	PACT (N = 473)		Non-PACT (N = 171)	PACT (N = 171)		
Demographics							
Age, years	65.64 ± 11.47	66.37 ± 11.19	0.223	68.20 ± 11.07	67.12 ± 10.54	0.353	0.101
Male, n (%)	1164 (76.2%)	350 (74.0%)	0.334	132 (77.2%)	134 (78.4%)	0.897	0.028
SBP (mmHg)	124.77 ± 21.34	127.39 ± 21.88	0.020	124.09 ± 22.12	124.15 ± 21.21	0.978	0.003
DBP (mmHg)	74.47 ± 13.13	76.33 ± 13.66	0.008	74.79 ± 13.84	75.27 ± 13.49	0.746	0.035
KILLIP classification, n (%)							
I	1058 (69.8%)	312 (67.2%)	0.183	122 (71.3%)	118 (69.0%)	0.896	0.084
II	347 (22.9%)	112 (24.1%)	​	35 (20.5%)	40 (23.4%)	​	​
III	75 (5.0%)	21 (4.5%)	​	11 (6.4%)	11 (6.4%)	​	​
IV	35 (2.3%)	19 (4.1%)	​	3 (1.8%)	2 (1.2%)	​	​
Comorbidities, n (%)							
Smoke	523 (34.3%)	151 (31.9%)	0.350	52 (30.4%)	56 (32.7%)	0.727	0.05
Hypertension	900 (58.9%)	288 (60.9%)	0.442	124 (72.5%)	115 (67.3%)	0.346	0.115
Diabetes	606 (39.7%)	194 (41.0%)	0.599	70 (40.9%)	74 (43.3%)	0.742	0.047
COPD	37 (2.4%)	10 (2.1%)	0.700	6 (3.5%)	5 (2.9%)	1	0.033
Previous MI	561 (36.7%)	50 (10.6%)	<0.001	29 (17.0%)	29 (17.0%)	1	<0.001
Prior-PCI	249 (16.9%)	43 (16.8%)	0.973	29 (17.0%)	27 (15.8%)	0.884	0.032
Previous stroke	111 (7.3%)	33 (7.0%)	0.832	14 (8.2%)	19 (11.1%)	0.464	0.099
Previous atrial fibrillation, n (%)	37 (2.4%)	22 (4.7%)	0.012	14 (8.2%)	15 (8.8%)	1	0.021
Laboratory measurements							
Albumin, mmol/L	35.36 ± 4.40	35.56 ± 4.48	0.392	33.93 ± 4.47	33.71 ± 3.81	0.625	0.053
LVEF (%)	52.94 ± 11.25	52.53 ± 10.66	0.503	51.63 ± 10.89	50.72 ± 11.44	0.453	0.081
Hemoglobin, g/L	134.43 ± 27.93	138.97 ± 39.35	0.020	135.45 ± 17.40	135.12 ± 16.57	0.858	0.019
Anemia, n (%)	524 (34.3%)	154 (32.8%)	0.507	50 (29.2%)	48 (28.1%)	0.905	0.026
Baseline creatinine, mg/dL	1.12 ± 0.75	1.08 ± 0.47	0.157	1.13 ± 0.43	1.17 ± 0.48	0.385	0.094
eGFR, mL/min	82.21 ± 30.17	84.63 ± 34.72	0.174	75.86 ± 25.05	75.41 ± 28.69	0.877	0.017
HbA1 c	6.71 ± 1.65	6.71 ± 1.76	0.611	6.86 ± 1.60	6.93 ± 1.86	0.711	0.04
Total cholesterol, mmol/L	4.82 ± 1.22	4.79 ± 1.25	0.680	4.80 ± 1.21	4.77 ± 1.18	0.805	0.027
LDL-C, mmol/L	3.17 ± 0.97	3.11 ± 0.98	0.242	3.04 ± 0.97	3.06 ± 0.97	0.865	0.018
HDL-C, mmol/L	0.99 ± 0.27	0.99 ± 0.28	0.774	1.01 ± 0.35	0.98 ± 0.30	0.393	0.092
Triglyceride, mmol/L	1.65 ± 1.13	1.47 ± 0.94	0.007	1.54 ± 1.15	1.50 ± 1.03	0.746	0.035
Medication use during hospitalization, n (%)							
Aspirin	1500 (98.2%)	455 (96.2%)	0.012	165 (96.5%)	168 (98.2%)	0.499	0.11
Clopidogrel	1333 (87.3%)	352 (74.4%)	<0.001	169 (98.8%)	167 (97.7%)	0.68	0.089
Statins	1504 (98.4%)	446 (94.3%)	<0.001	167 (97.7%)	166 (97.1%)	1	0.037
Betablocker	1239 (81.1%)	349 (73.8%)	<0.001	150 (87.7%)	151 (88.3%)	1	0.018
Insulin	302 (19.8%)	99 (20.9%)	0.580	33 (19.3%)	38 (22.2%)	0.594	0.072
ACEI/ARB	1207 (79.0%)	380 (80.3%)	0.528	147 (86.0%)	148 (86.5%)	1	0.017
Calcium channel blocker	171 (11.2%)	53 (11.2%)	0.993	22 (12.9%)	26 (15.2%)	0.64	0.067
GP IIb/IIIA inhibitor	532 (34.9%)	213 (45.1%)	<0.001	98 (28.7%)	73 (21.4%)	0.742	​
Procedural characteristics							
Treated_lession, n (%)							
Left anterior descending artery	542 (35.47%)	193 (40.38%)	0.003	72 (42.11%)	60 (35.09%)	0.063	​
Left circumflex artery	148 (9.69%)	40 (8.37%)	​	14 (8.19%)	22 (12.87%)	​	​
Right coronary artery	485 (31.74%)	166 (34.73%)	​	52 (30.41%)	68 (39.77%)	​	​
Multi coronary artery	350 (22.91%)	73 (15.27%)	​	32 (18.71%)	21 (12.28%)	​	​
Multi-vessel stenosis, n (%)	1361 (89.1%)	333 (70.4%)	<0.001	141 (82.5%)	138 (80.7%)	0.78	0.045
Radial artery access site, n (%)	1368 (89.6%)	422 (89.2%)	0.071	​	​	​	​
Femoral artery access site, n (%)	158 (10.4%)	51 (10.8%)	​	​	​	​	​
Number of stents	1.61 ± 2.66	1.60 ± 1.87	0.918	1.45 ± 0.90	1.40 ± 0.72	0.596	0.057
Length of stent (mm)	40.93 ± 24.37	40.23 ± 22.42	0.566	36.26 ± 24.06	35.44 ± 19.82	0.73	0.037
Contrast volume (mL)	117.39 ± 40.58	132.85 ± 49.53	<0.001	125.47 ± 45.08	123.25 ± 42.25	0.638	0.051
Length of hospital stay, days	7.00 (5.00–8.00)	7.00 (6.00–9.00)	0.028	​	​	​	​

PACT: parenteral anticoagulation therapy; SBP: systolic blood pressure; DBP: diastolic blood pressure; COPD: chronic obstructive pulmonary disease; MI: myocardial infarction; PCI: percutaneous coronary intervention; LVEF: left ventricular ejection fraction; WBC: white blood cell; eGFR: estimated glomerular filtration rate; HbA1c: glycosylated hemoglobin; LDL-C: low-density lipoprotein cholesterol; HDL-C: high-density lipoprotein cholesterol; ACEI/ARB: angiotensin converting enzyme inhibitor/angiotensin receptor blocker.

Subgroup analyses of primary outcomes were performed based on age (<65 years or ≥65 years), sex (male or female), diabetes mellitus (yes or no), hypertension (yes or no), and renal insufficiency (yes or no). The interaction P value was obtained by adding a multiplicative interaction term. All statistical analyses were performed using SAS 9.4 (SAS Institute, Cary, NC), with a threshold of P < 0.05 to determine statistical significance.

## 3 Results

### 3.1 Baseline characteristics

A total of 5,335 patients with STEMI who underwent PCI were enrolled, and 2,001 patients were finally included. Among the analyzed cohort, 473 patients (23.64%) received PACT, while 1,528 (76.36%) did not (non-PACT) (Figure 1). Gender distribution was similar between the two groups. Compared to the non-PACT group, the PACT group consisted of younger patients with higher systolic and diastolic blood pressure, and a lower rate of previous myocardial infarction, atrial fibrillation, and hypertension (Table 1).

**FIGURE 1:**
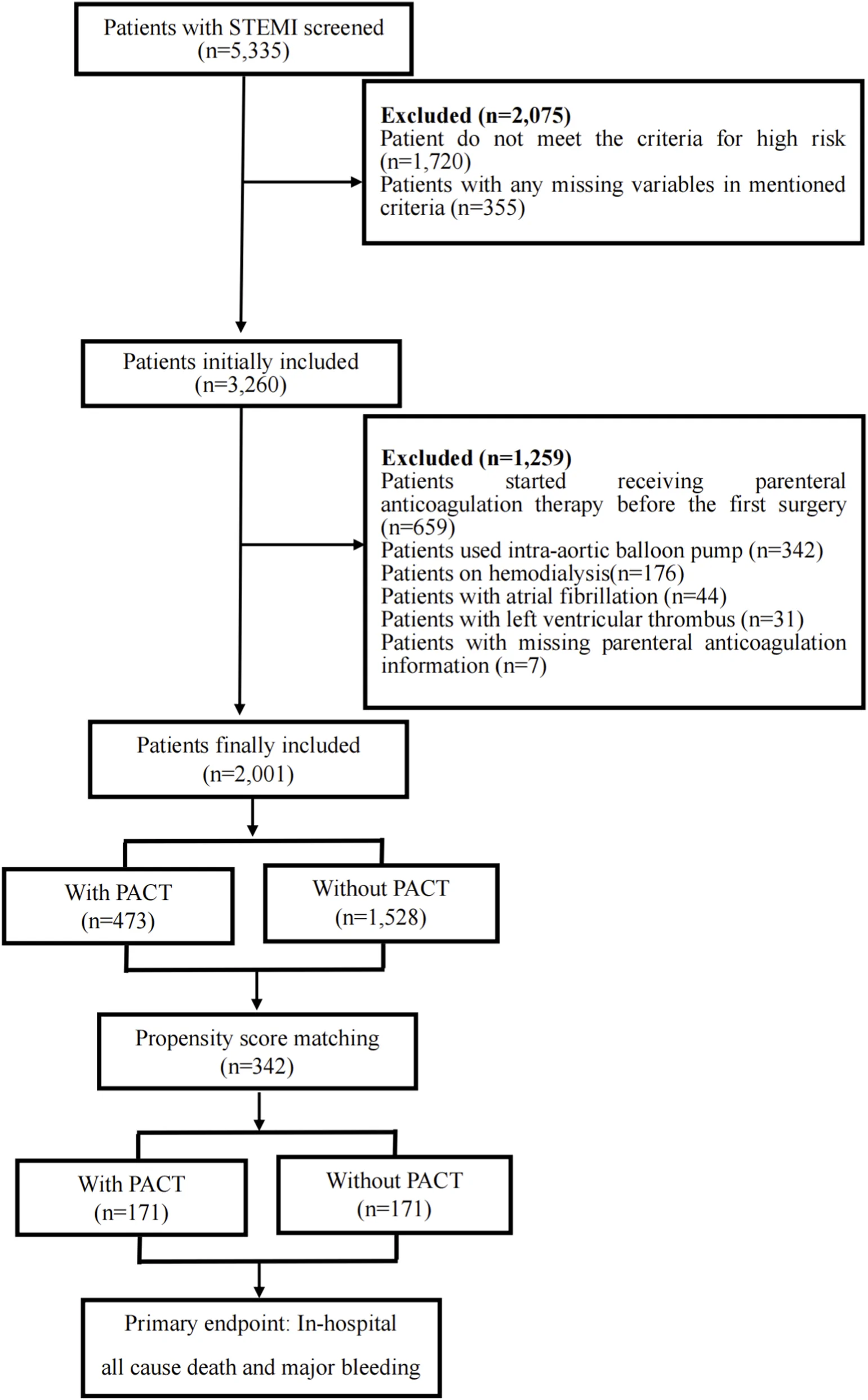
Study flow chart. Note: STEMI: ST-segment elevation myocardial infarction; PACT: parenteral anticoagulation therapy.

Laboratory results showed significantly higher hemoglobin levels and lower triglyceride levels in the PACT group. Moreover, patients in the PACT group were administered less aspirin, clopidogrel, statins, and beta-blockers. However, they received a higher volume of contrast media, had extended hospital stays, and a lower incidence of multivessel stenosis. Radial access was used in the majority of patients (PACT group: 89.6% vs. non-PACT group: 89.2%; P = 0.071), with no significant difference between groups (Table 1).

### 3.2 Clinical outcomes during hospitalization

There was no significant difference in in-hospital all-cause death (2.1% for the PACT group vs. 2.3% for the non-PACT group; P = 0.821) or major bleeding (11.8% for the PACT group vs. 13.5% for the non-PACT group; P = 0.637). Additionally, other clinical outcomes were comparable between the PACT and non-PACT groups (Table 2). Multivariable analyses indicated that PACT was not linked to a reduced risk of in-hospital all-cause death (adjusted odds ratio [OR], 0.70; 95% confidence interval [CI], 0.33–1.48; P = 0.349) or major bleeding (adjusted OR, 0.84; 95% CI, 0.32–2.18; P = 0.720). Similar findings were observed for other clinical outcomes (Table 3). And there was not significantly different between the PACT and non-PACT groups in thrombotic events (Supplementary Table S1). Additionally, the time-varying Cox analysis produced estimates in the same direction as the primary logistic regression analysis and did not materially alter the statistical inference (hazard ratio [HR] for in-hospital death, HR = 0.65, 95%CI = 0.30–1.41, P = 0.274; HR for major bleeding, HR = 0.62, 95%CI = 0.10–3.74, P = 0.604 (Supplementary Table S2), and the primary results were unchanged.

**TABLE 2:** Clinical outcomes.

Outcomes, n (%)	Total (n = 2001)	Without parenteral anticoagulation therapy (N = 1,528)	Parenteral anticoagulation therapy (N = 473)	*P* value
In hospital				
Death	45 (2.2)	35 (2.3)	10 (2.1)	0.821
MACEs	73 (3.6)	59 (3.9)	14 (3.0)	0.361
Any bleeding	289 (14.4)	227 (14.9)	62 (13.1)	0.334
Major bleeding	262 (13.1)	206 (13.5)	56 (11.8)	0.637
NACEs	96 (4.8)	76 (5.0)	20 (4.2)	0.507
Follow up death				
One year	100 (5.0)	81 (5.3)	19 (4.0)	0.263
Three year	147 (7.3)	103 (6.7)	44 (9.3)	0.062
Five year	161 (8.0)	113 (7.4)	48 (10.1)	0.054
Follow-up outcomes*				
One year	114 (5.7)	94 (6.2)	20 (4.2)	0.115
Three year	169 (8.4)	122 (8.0)	47 (9.9)	0.182
Five year	183 (9.1)	132 (8.6)	51 (10.8)	0.158

MACEs, indicate major adverse cardiovascular events; NACEs, indicate net adverse clinical events

* Follow-up outcomes included all cause death and stroke.

**TABLE 3:** Analyses of different outcomes between PACT and non-PACT group.

Outcomes	Univariate analysis			Multivariate analysis*			Propensity score matching analysis		
	OR	95% CI	*P* value	OR	95% CI	*P* value	OR/HR	95% CI	*P* value
In hospital									
Death	0.92	0.45–1.87	0.821	0.70	0.33–1.48	0.349	0.56	0.16–1.95	0.364
MACEs	0.76	0.42–1.37	0.363	0.64	0.35–1.20	0.168	0.52	0.20–1.33	0.173
Major bleeding	0.92	0.37–2.29	0.857	0.84	0.32–2.18	0.720	1.51	0.25–9.15	0.655
Any bleeding	0.86	0.64–1.17	0.335	0.81	0.59–1.11	0.182	1.18	0.67–2.01	0.570
NACEs	0.84	0.51–1.40	0.508	0.73	0.43–1.24	0.242	0.70	0.30–1.62	0.399
Follow up									
Death	0.92	0.65–1.29	0.629	0.94	0.67–1.33	0.747	0.97	0.51–1.86	0.931
Outcomes^#^	1.02	0.73–1.41	0.924	1.03	0.74–1.43	0.873	0.97	0.52–1.79	0.918

MACEs, indicate major adverse clinical events; NACEs, indicate net adverse clinical events; OR, indicates odds ratio. #Outcomes included all cause death and stroke.

* adjusted age, gender, smoke, diabetes, hypertension, prior myocardial infarction, prior stroke, chronic obstructive pulmonary disease, anemia, PCI access site, estimated glomerular filtration rate.

### 3.3 Follow up clinical outcomes

At a median follow-up of 1.72 years (interquartile range, 0.04–3.05 years), the incidence of all-cause death (adjusted HR, 0.94; 95% CI, 0.67–1.33; P = 0.747) and MACEs (adjusted HR, 1.03; 95% CI, 0.74–1.43; P = 0.873) were comparable between the PACT and non-PACT groups (Table 3; Figures 2A,B).

**FIGURE 2:**
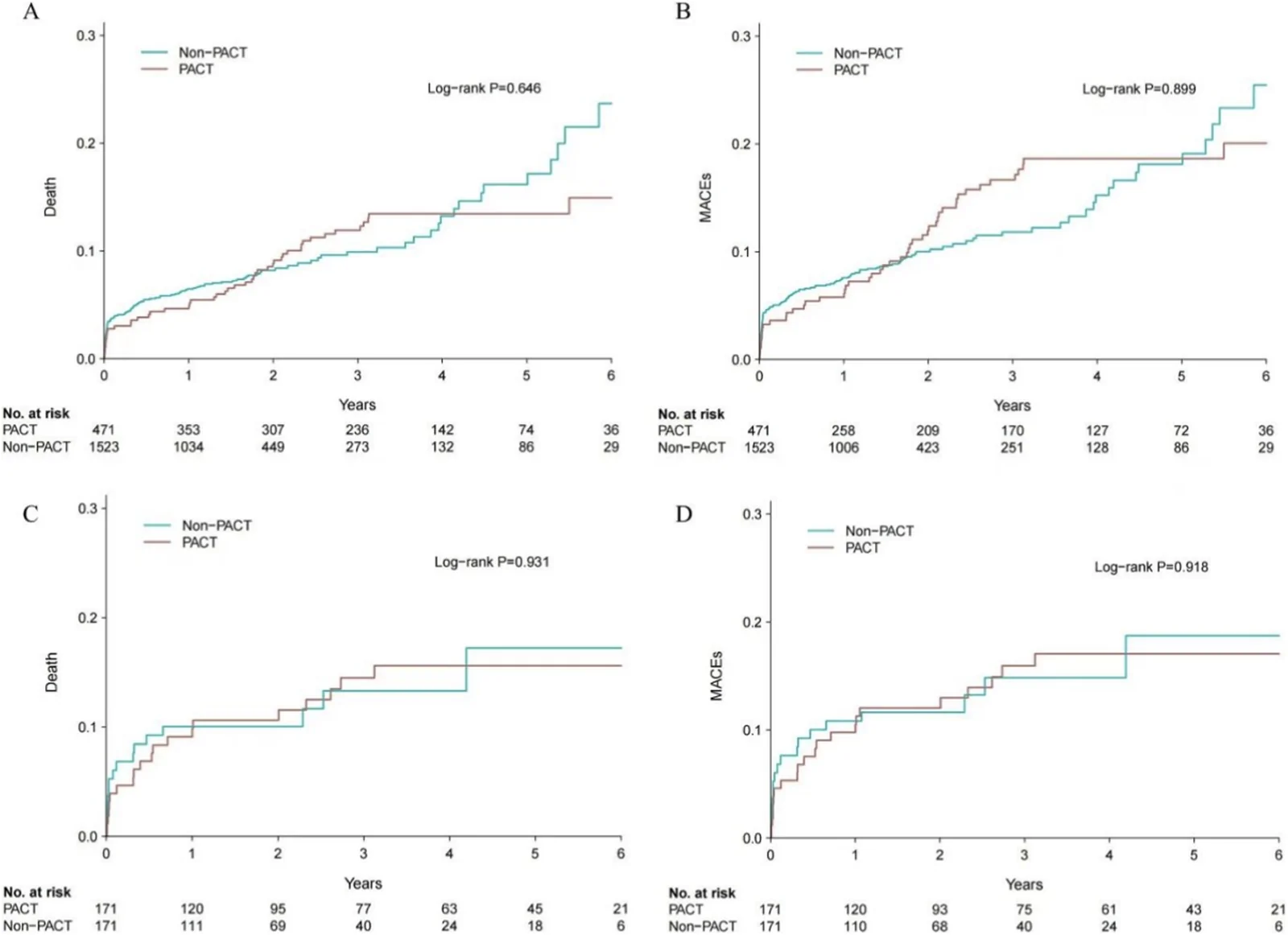
Kaplan-Meier curves of all-cause death and major adverse cardiovascular events. Note: PACT, parenteral anticoagulation therapy; MACEs, major adverse clinical events.

### 3.4 Propensity score analyses

We matched 171 patients from the PACT group to 171 patients from the non-PACT group in a 1:1 ratio. This matching process eliminated differences in baseline characteristics between the groups, with all variables displaying a standardized difference of less than 10% (Table 1; Figure 3). Post-matching results showed no significant difference in in-hospital all-cause death (OR, 0.56; 95% CI, 0.16–1.95; P = 0.364) or in-hospital major bleeding (OR, 1.51; 95% CI, 0.25–9.15; P = 0.655) between the PACT and non-PACT groups (Table 3). Similarly, the risk of all-cause death and MACEs during follow-up was comparable between the two groups (Table 3; Figures 2C–D).

**FIGURE 3:**
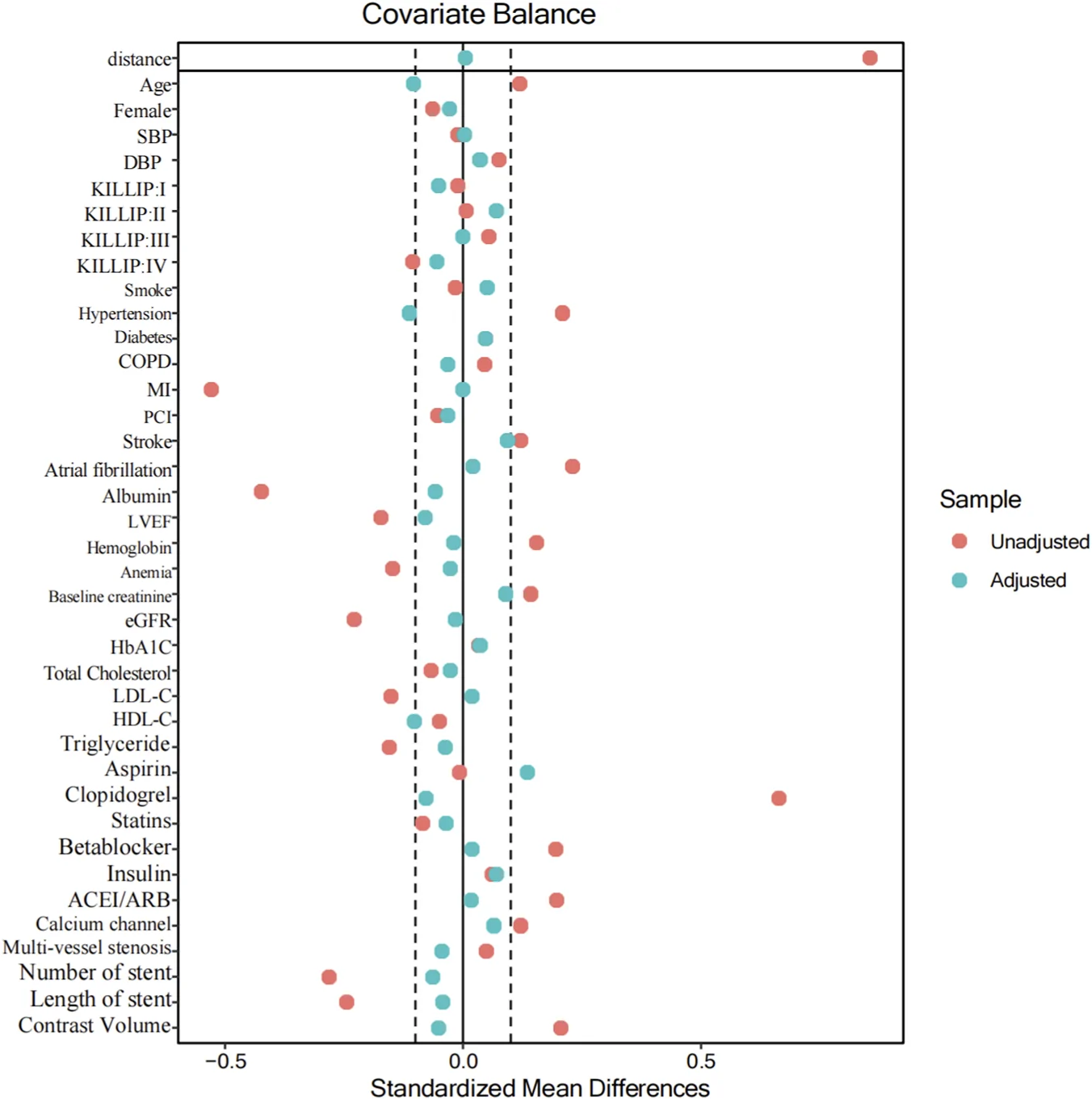
A “love” plot graphically displaying the covariate balance before and after adjustment.

### 3.5 Subgroup analysis

The effect of PACT on in-hospital clinical outcomes did not significantly differ across various subgroups (Supplementary Figures S1-S5). The interaction p-values for these subgroup analyses did not indicate significant heterogeneity in the effects of PACT across these groups.

## 4 Discussion

The present study might be the first to show that PACT after PCI was not associated with a reduced risk of ischemic events or increased risk of bleeding in STEMI patients with high thrombotic risk.

Antiplatelet therapy remains the foundational treatment for STEMI patients, and its combination with anticoagulation before and during PCI is recommended by current guidelines to reduce ischemic events (Ibanez et al., 2018; Oldgren et al., 2008; Cantor et al., 2020; Kapil et al., 2017). For patients with acute coronary syndrome, low-dose rivaroxaban in combination with dual antiplatelet therapy reduced the risk of stent thrombosis after PCI by one-third but tripled the risk of major bleeding (Mega et al., 2012). A previous randomized controlled trial involving over 6,000 patients found that compared to heparin monotherapy, anticoagulation with bivalirudin plus a post-PCI high-dose infusion for 2–4 h significantly reduced 30-day all-cause mortality without increasing major bleeding (Li et al., 2022).

However, recent research has demonstrated that routine postprocedural anticoagulation after primary PCI in STEMI patients is safe but does not reduce 30-day ischemic events (Yan et al., 2024). The latest guidelines advise against the routine use of PACT after revascularization, except in cases involving clear indications such as ventricular thrombus, slow- or no-reflow, atrial fibrillation, or other thrombotic complications (Ibanez et al., 2018; Chen et al., 2020). Given the lack of evidence of benefit from routine use, selecting the optimal STEMI patients who might benefit from anticoagulation therapy is crucial in reducing ischemic events. STEMI patients with high thrombotic risk have been shown to have a higher risk of ischemic events such as stent thrombosis and recurrent myocardial infarction (Antithrombotic Trialists, 2002; Baber et al., 2019). Combining anticoagulation with antiplatelet therapy may benefit such patients, although this remains unclear (Arishta Jhagroe and Maria Ten Berg, 2015).

A frequent limitation of previous studies is the absence of classification based on thrombosis risk, which may obscure the potential benefits of PACT by increasing the incidence of bleeding events in patients with low thrombosis risk. One study previously found that a higher dose of bivalirudin monotherapy with extended infusion post-stenting is safe and may play a crucial role in preventing stent thrombosis without raising bleeding events (Wang et al., 2017). However, this efficacy is more attributed to the pharmacological properties of bivalirudin rather than the strategy of prolonging anticoagulant therapy with agents like heparin. Therefore, our study is the first to find that PACT after PCI is not associated with reducing risk of ischemic events or an increasing risk of bleeding in STEMI patients with high thrombotic risk. It is not surprising that other studies have similarly concluded that warfarin does not reduce the risk of reinfarction, all-cause mortality, or stroke in STEMI patients post-PCI, while significantly elevating the risk of major bleeding (Le May et al., 2015). Likewise, recent research by Yan et al. did not demonstrate any positive impact of PACT on 30-day ischemic events (Yan et al., 2024).

In line with our previous research, PACT administered before revascularization did not improve outcomes in patients with non-ST elevation acute coronary syndrome. While high-risk patients typically face worse clinical outcomes, and approaches such as early coronary revascularization and intensive antiplatelet therapy effectively prevent ischemic events and thrombus in these individuals, the benefits of PACT may be masked by these treatments (Ibanez et al., 2018; Singh et al., 2018). Our findings support tightening current guidelines that discourage routine use of PACT after PCI, even in patients with high ischemic risk, no clinical benefit was observed.

Our findings align with recent studies indicating that PACT after PCI was not associated with an increased risk of bleeding (Yan et al., 2024; Yan et al., 2022). However, Ducrocq et al. reported that PACT after PCI is linked to higher rates of bleeding, including major and minor bleeding. This discrepancy might be due to a higher usage of glycoprotein IIb/IIIa inhibitors and a greater prevalence of femoral arterial access in their study, both of which are associated with increased bleeding risk, compared to our current research. While our study confirms that PACT after PCI is safe, it is important to note that this approach is not recommended even for STEMI patients with high thrombotic risk, as it does not show clear benefits in reducing ischemic events. And the high thrombotic risk definition was adopted from the TWILIGHT criteria and includes features—such as older age, female sex, and chronic kidney disease—that predict both ischemic and bleeding events. Our cohort therefore represents a population in which thrombotic and bleeding risks coexist, and the findings should be interpreted accordingly.

## 5 Limitations

First, as this was an observational study, potential selection bias and confounding factors could not be avoided. For example, the variability of follow-up, and the choice of treatment depended on the judgment of physicians. Despite applying many statistical methods, some relevant confounding factors may have remained unaddressed. Therefore, randomized controlled trials are needed to verify the efficacy and safety of PACT after PCI in STEMI patients with high thrombotic risk. Second, only STEMI patients were classified as high thrombotic risk were enrolled, which might underestimate the efficacy of PACT among patients with non-ST elevation acute coronary syndrome. Third, details on other procedural factors potentially affecting bleeding outcomes, such as sheath size and closure technique, was not collected during the study period, which may introduce potential confounding effects. Forth, while propensity score matching reduced the sample to 171 pairs, the primary multivariable analyses retained the full cohort and showed consistent null results. Nevertheless, the reduced matched sample limits the precision of effect estimates in this sensitivity analysis. Finally, patients with indications for anticoagulant therapy after PCI, such as those with atrial fibrillation were excluded and the obtained results might not apply to this subset of patients.

## 6 Conclusion

In this multicenter cohort of STEMI patients with high thrombotic risk, post-PCI parenteral anticoagulation was not associated with a statistically significant reduction in in-hospital mortality or ischemic events, nor was it associated with a statistically significant increase in major bleeding. These findings support current guideline recommendations against routine PACT use but should not be interpreted as evidence of equivalence or non-inferiority, given the observational design and wide confidence intervals.

## Data Availability

The original contributions presented in the study are included in the article/Supplementary Material, further inquiries can be directed to the corresponding author.
