# Supplementary material for: Association between post procedural parenteral anticoagulation therapy and clinical outcomes in ST-elevation myocardial infarction patients with high thrombotic risk
Source: Front Pharmacol. 2026 Sep 22;17:1747506. doi: 10.3389/fphar.2026.1747506 (PMC13639302; doi:10.3389/fphar.2026.1747506)
Supplement: Supplementary file 1 [file Supplementaryfile1.doc]

**Figure S1.** Subgroup analysis for in-hospital all-cause death.

**Figure S2.** Subgroup analysis for in-hospital major adverse cardiovascular events.

**Figure S3.** Subgroup analysis for in-hospital net adverse clinical events.

**Figure S4.** Subgroup analysis for in hospital major bleeding.

**Figure S5.** Subgroup analysis for in hospital any bleeding.

| **Outcomes, n (%)** | **Total**  **(n=2001)** | **Without Parenteral Anticoagulation Therapy(N=1528)** | **Parenteral Anticoagulation Therapy(N=473)** | ***P* value** |
| --- | --- | --- | --- | --- |
| Stroke | 18 (0.9) | 16 (1.0) | 2 (0.4) | 0.212 |
| Revascularization | 13 (0.65) | 11 (0.7) | 2 (0.4) | 0.487 |
| Re-myocardial infarction | 8 (0.4) | 6 (0.4) | 2 (0.4) | 0.928 |

**Supplemental Table1**. Univariate analysis of clinical outcomes.

| **Outcomes** | **Cox analysis** | | |
| --- | --- | --- | --- |
|  | **OR** | **95% CI** | ***P* value** |
| **In hospital** |  |  |  |
| Death | 0.65 | 0.30-1.41 | 0.274 |
| MACEs | 0.54 | 0.29-1.02 | 0.057 |
| Any bleeding | 0.61 | 0.40-0.94 | 0.026 |
| Major bleeding | 0.62 | 0.10-3.74 | 0.604 |
| **Follow up** |  |  |  |
| Death | 0.97 | 0.68-1.37 | 0.850 |
| Outcomes | 1.07 | 0.77-1.49 | 0.693 |

**Supplemental Table 2.** Time-dependent Cox regression model of outcomes.

Note: MACEs indicate major adverse clinical events; OR indicates odds ratio; Outcomes included all cause death and stroke.
